# Case for diagnosis. Hair analysis in a child with delayed psychomotor development and fragile and brittle hair: Trichothiodystrophy^☆^

**DOI:** 10.1016/j.abd.2021.10.015

**Published:** 2022-12-14

**Authors:** Irene López Riquelme, Alberto Andamoyo Castañeda, Elisabeth Gómez Moyano, Ángel Vera Casaño

**Affiliations:** Department of Dermatology, Hospital Regional Universitario de Málaga, Málaga, Spain

Dear Editor,

A 4-year-old female child presented to the Dermatology Department with short, thin, and fragile hair since birth (Fig. 1). She also presented important xerosis and eczematous plaques in her back, trunk, and scalp and photosensitivity. The patient also had short stature, severe myopia, delayed psychomotor development, and recurrent respiratory infections.

**Figure 1:**
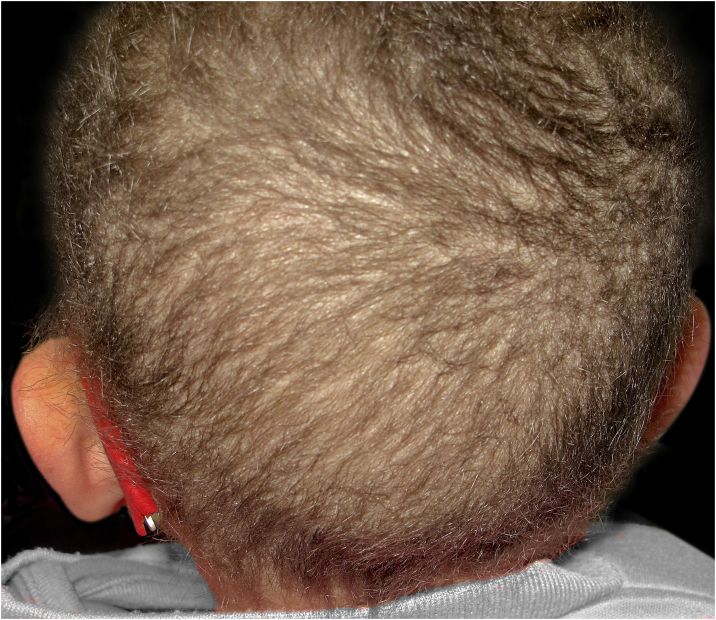
Short brittle hair easily broken at different lengths.

Examination of the hair under a polarized light microscope showed fine hair and trichoscisis with typical alternating dark and light transverse banding, called “tiger-tail pattern” (Fig. 2) and an irregular surface (Fig. 3).

**Figure 2:**
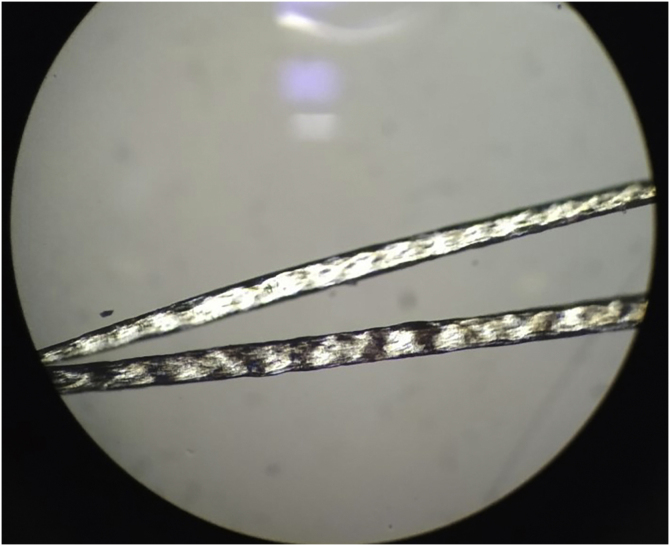
Examination of the hair under a polarized microscope: trichoscisis with typical “tiger-tail pattern”. Note the alternating dark and light transverse banding.

**Figure 3:**
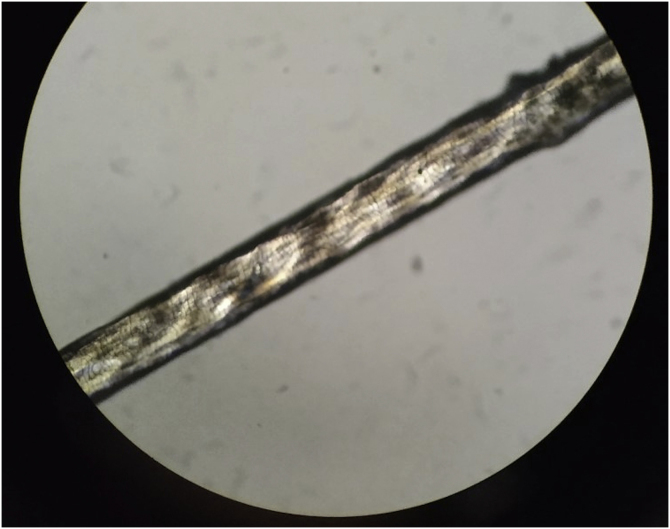
Irregular, undulating hair shaft when examined under light microscopy.

A genetic study revealed a mutation in the ERCC2 gene.

## What is your diagnosis?

a) Xeroderma Pigmentosum
b) Trichothiodystrophy
c) Menkes disease
d) Cockayne syndrome

## Discussion

Based on clinical presentation, trichological and genetic examination, the diagnosis of trichothiodystrophy was established.

Trichothiodystrophy (TTD) is a heterogeneous group of neuroectodermal disorders with an autosomal recessive inheritance, although a few cases with possible X-linked transmission have been reported. The photosensitive form of TTD is caused by mutations in XPB, XPD, or p8/TTDA genes, which encode subunits of TFIIH transcription/repair factor. Non-photosensitive form of TTD is genetically heterogeneous, being TTDN1 gene the one described in a small proportion of patients.^1^ In photosensitive TTD, the most frequently described is XPD (ERCC2) mutation,^2^ which is also involved in the pathogenesis of xeroderma pigmentosum (XP) and Cockayne syndrome, although, unlike XP, there is no predisposition to cutaneous malignancies. XP, Cockayne syndrome, and TTD are an example of the phenomenon called clinical heterogeneity, in which mutations in one gene (in this case XPD) may result in distinct diseases or variants.^3^

Clinical features of patients with TTD vary widely in nature and severity, and the single common feature in all patients is fragile hair (short, unruly, fragile hair of the scalp, eyebrows, and eyelashes) due to abnormally low sulfur content. In addition, a wide spectrum of other clinical symptoms that usually affect organs of ectodermal and neuroectodermal origin may be present, such as intellectual and growth retardation, ichthyosis, short stature, decreased fertility, neurologic and ocular abnormalities and, in some cases, recurrent infections,^4^ as in the case of our patient. Approximately half of the patients present photosensitivity.^1^, ^4^

When examined under a polarized microscope, hair samples constantly show striking bright and dark transverse banding or “tiger tail pattern”, and they often exhibit an undulating, irregular contour in all hairs (differently from pseudo-tiger tail banding).^5^, ^6^ Trichoschisis and trichorrhexis nodosa-like defects are also distinctive hair shaft abnormalities in TTD though not always present. In contrast to TTD, other patients with similar defects in DNA repair and mutations in the XPD gene do not show true “tiger tail banding”. A “pseudo-tiger tail banding” can be observed in segments of normal shafts, but the banding pattern is usually less pronounced and less regular than the bright and dark banding observed in TTD patients.^5^ In fact, characteristic microscopic hair findings distinguish trichothiodystrophy from other conditions with congenital alopecia or hypotrichosis. For example, patients with Menkes disease typically present “kinky hairs” with twists around their long axis at irregular intervals in the shaft when observed under a light microscope, also known as pili torti.^7^

In conclusion, analysis of the hair under a polarized microscope is considered a very useful diagnostic marker in trichothiodystrophy since it shares clinical and genetic characteristics with other neuroectodermal syndromes. Diagnosis can be made on the basis of clinical and trichological examination with a polarizing and light microscope, although a genetic study may be helpful.

## Financial support

None declared.

## Authors' contributions

Irene López Riquelme: Critical review of the literature; writing of the manuscript; final approval of the final version of the manuscript.

Alberto Andamoyo Castañeda: Critical review of the literature; writing of the manuscript; final approval of the final version of the manuscript.

Elisabeth Gómez Moyano: Intellectual participation in the propaedeutic conduct of the case; final approval of the final version of the manuscript.

Ángel Vera Casaño: Intellectual participation in the propaedeutic conduct of the case; final approval of the final version of the manuscript.

## Conflicts of interest

None declared.
